# Application Research of Three-Dimensional Printing Technology and Three-Dimensional Computed Tomography in Segmentectomy

**DOI:** 10.3389/fsurg.2022.881076

**Published:** 2022-04-29

**Authors:** Li Tongxin, Xu Jing, Wang Runyuan, Wu Wei, Zhou Yu, Wang Dong, He Wang, Wu Yi, He Ping, Fu Yong

**Affiliations:** ^1^ Clinical Medicine Department, North Sichuan Medical College, Nanchong, Sichuan, China; ^2^ Cardiothoracic Surgery Department, Dianjiang People’s Hospital of Chongqing, Chongqing, China; ^3^ Health Economy Department, Southwest Hospital, Chongqing, China; ^4^ Thoracic Surgery Department, Southwest Hospital, Chongqing, China; ^5^ Cardic Surgery Department, Southwest Hospital, Chongqing, China; ^6^ Digital Medicine Department, Biomedical Engineering College, Army Military Medical University, Chongqing, China

**Keywords:** segmentectomy, 3D printing technology, 3D computed tomography, lung cancer, pulmonary nodules

## Abstract

**Background:**

To compare the application of the emerging 3D printing technology and 3D-CT in segmentectomy. And to explore the advantages of 3D printing technology in thoracoscopic segmentectomy.

**Methods:**

We collected the clinical data of 118 patients undergoing thoracoscopic segmentectomy from January 2019 to April 2021 at the Thoracic Surgery Department, the Dianjiang People's Hospital of Chongqing and Southwest Hospital. Among them, 61 patients were in the 3D printing group and 57 patients were in the 3D-CT group respectively. The perioperative data of these two groups of patients were analyzed respectively.

**Results:**

There were no significant differences between the two groups in age, gender, tumor diameter, pathology, the preoperative complications of diabetes and heart disease. However, the patients with the complications of hypertension in the 3D printing group are significantly more than the 3D-CT group (*P* = 0.003). Compared with the 3D-CT group, patients in the 3D printing group had significantly shorter operation time (162.7 ± 47.0 vs. 190.3 ± 56.9 min, *P* = 0.006), less intraoperative fluid input (1,158.5 ± 290.2 vs. 1,433.2 ± 653.3, *P* = 0.013), and less total intraoperative fluid output, including intraoperative blood loss, urine excretion, and other fluid loss. In addition, there were no statistically significant differences in intraoperative blood loss, 24 h pleural fluid volume, 48 h pleural fluid volume, postoperative chest tube duration, postoperative hospital stay and complications between the two groups of patients (*P* > 0.05).

**Conclusions:**

In thoracoscopic segmentectomy, the application of 3D printing technology shortens the operation time, reduces intraoperative fluid input and output, guides the operation more safely and effectively, and has better clinical application value.

## Introduction

With the widespread application of low-dose computed tomography (CT) in physical examinations, more and more cases with early non-small cell lung cancer (NSCLC) have been reported (1). At present, the standard surgical treatment for early NSCLC is lobectomy plus lymph node dissection (2). However, a randomized controlled trial involving 1,106 people showed that except for more air leakage in the segmentectomy group, there are no differences in intraoperative and postoperative complications and any other postoperative indicators between the segmentectomy group and lobectomy group (3), which is why more and more hospitals choose segmentectomy for patients with early NSCLC. However, segmentectomy has greater risks than lobectomy mainly due to the complex anatomical structures of the lung segments and the high variability of blood vessels and bronchi. Therefore, preoperative planning is particularly important. Currently, the 3D-CT is one of the most widely used preoperative modeling technique in clinical practice and has been widely used in various clinical disciplines.

In thoracic surgery, 3D-CT is used to reconstruct 3D images of the blood vessels, bronchi and tumors of the target lung lobes. The 3D images of the blood vessels, bronchi, and tumors of the diseased lung lobes can be well reconstructed, which is of great significance for performing thoracoscopic surgery efficiently and safely (4). However, displacing the 3D images on a two-dimensional screen limits the judgment of surgeons on the relative position of tumors and the target lung segments. Therefore, a new preoperative modeling method needs to be established to make up for this deficiency.

3D printing is a manufacturing method that originated in the 1980s (5). It has been used in cardiac surgery, breast surgery, ear, nose, and throat (ENT), and other clinical disciplines. Also it is considered to have certain clinical value (6–8). At present, there are few reports on the application of 3D printing technology in thoracic surgery. Studies have applied 3D printing models to the preoperative positioning of small pulmonary nodules and shown that it can locate small pulmonary nodules faster and more accurately and reduce the time of exposing patients to radiation than the traditional 3D-CT (9, 10). To compare the clinical application values of 3D printing technology and 3D-CT, this study applied 3D-CT and 3D printing technology to the preoperative planning of thoracoscopic segmentectomy and compared the perioperative indicators of patients in the 3D printing group and 3D-CT group.

## Methods

### Patient Selection Criteria

The study is a retrospective analysis of the clinical data of 118 patients undergoing thoracoscopic segmentectomy from January 2019 to April 2021 in the Dianjiang People's Hospital of Chongqing and Southwest Hospital. Among them, 20 patients were from the Dianjiang People's Hospital of Chongqing and 98 patients were from the Southwest Hospital. There are two skilled medical groups in each of the two hospitals. Each group receives patients and operates in turn. In each hospital, one group chose 3D printing technology for preoperative planning, and the other group chose 3D CT. All enrolled patients strictly followed a consistent preoperative planning procedure, and all enrolled patients were treated with the same surgical instruments and thoracoscopy. There were no differences between facilities in patient distribution. Among the 118 patients, 61 patients were in the 3D printing group and 57 patients in the 3D-CT group. According to the NCCN guidelines (11), patients who met the following criteria were enrolled: (1) patients with poor lung functions or unable to tolerate lobectomy due to other severe comorbidities, (2) patients with peripheral nodules with a diameter ≤2.0 cm and with either simple carcinoma in situ or ≥50% ground glass component of the nodule in CT, and (3) the nodule doubling time >400 d as confirmed by imaging examination and monitoring.

### Preoperative 3D Image Construction and 3D Model Printing

**Figure 1:**
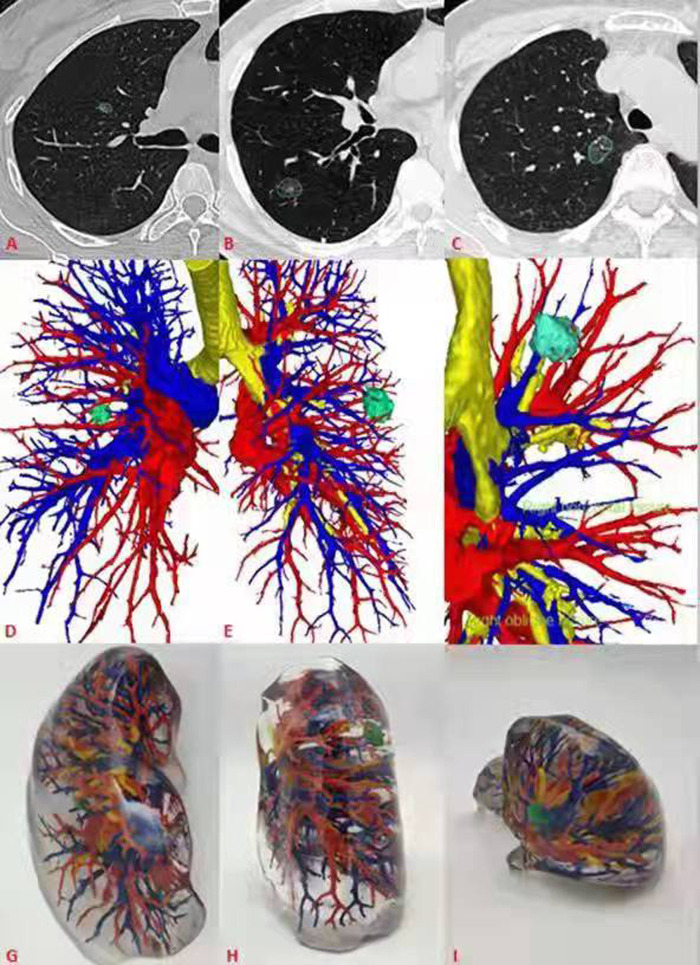
General CT, 3D-CT and 3D printing models of 3 patients. (**A**–**C**) General CT showed ground glass nodules in the anterior segment of the right upper lung, back segment of the right lower lung, and posterior segment of the right upper lung respectively. (**D**–**F**) 3D-CT showed the relationship between pulmonary nodules and peripheral bronchi and pulmonary vessels. (**G**–**I**) The 3D printing model shows the pulmonary nodules and its relationship.

**Figure 2:**
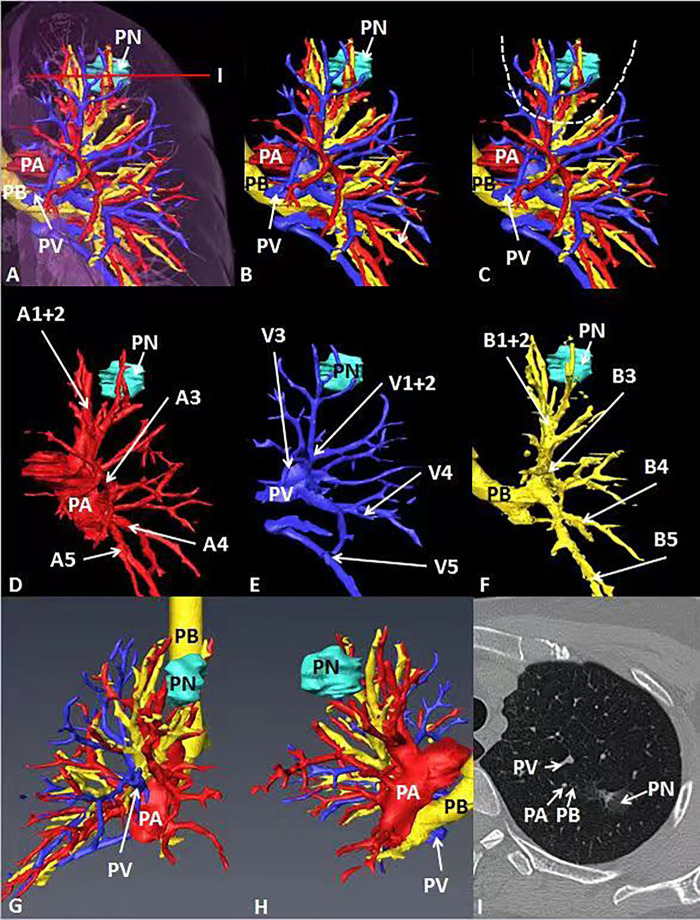
General CT, 3D-CT of the patient. (**A–C**) 3D-CT of the patient shows the location of pulmonary nodules in the left upper lung. (**D–F**) 3D-CT shows the position between target pulmonary nodules and surrounding pulmonary arteries, veins and bronchi. (**G, H**) Observe the position between target pulmonary nodules and surrounding pulmonary arteries, veins and bronchi from different perspectives. (**I**) The position between the target pulmonary nodule and the surrounding pulmonary artery, vein and bronchus on the General CT.

**Figure 3:**
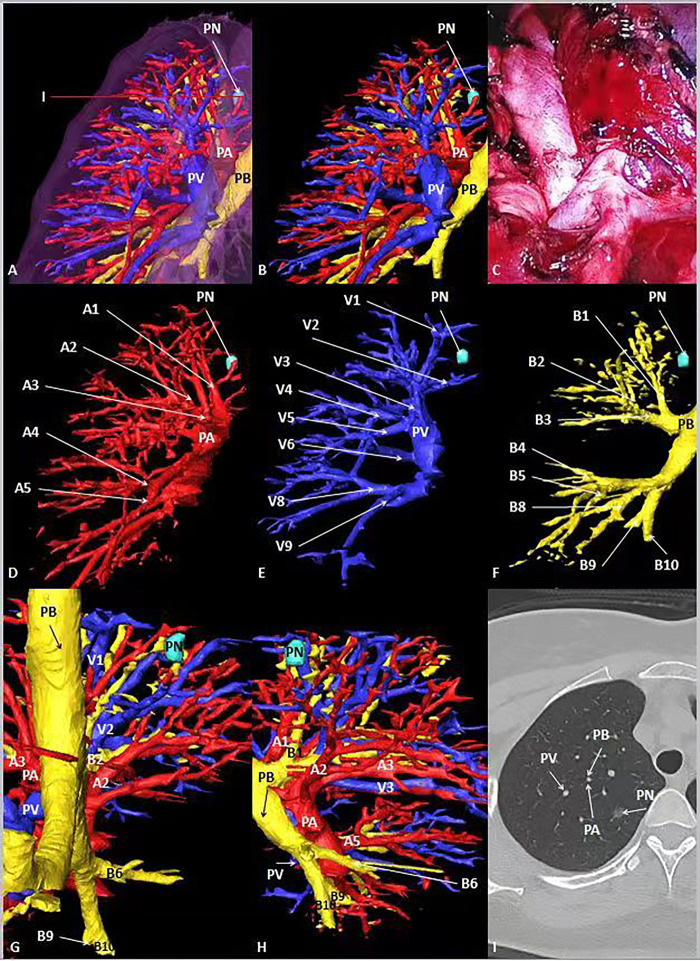
General CT, 3D-CT of the patient. (**A**, **B**) 3D-CT of the patient shows the location of pulmonary nodules in the right upper lung. (**C**) Intraoperative condition. (**D**–**F**) 3D-CT shows the position between target pulmonary nodules and surrounding pulmonary arteries, veins and bronchi. (**G**, **H**) Observe the position between target pulmonary nodules and surrounding pulmonary arteries, veins and bronchi from different perspectives. (**I**) The position between the target pulmonary nodule and the surrounding pulmonary artery, vein and bronchus on the General CT.

The preoperative CT data of patients in the 3D printing group and 3D-CT group were from the Imaging Department, the Dianjiang People's Hospital of Chongqing and the Southwest Hospital. The DICOM data of the thin-layer (0.625–1.25 mm) CT images were imported into Mimics software (vision 21.0). The lung cancer and its adjacent pulmonary segmental artery, vein, bronchus were segmented and 3D reconstructed with Mimics software.Then, the 3D model was imported to the liquid photosensitive resin printer in the STL format. After printing, the model surface was smoothed, and the tumors, pulmonary arteries, veins, bronchi and parenchyma in the model were colored in green, red, blue. yellow and transparent. At last, the best surgical approach was determined by measuring the distance of the tumor to the nearby blood vessels and bronchi (**Figures 1–3**).

### Operation Procedure

All patients were positioned at the lateral position and ventilated with two chambers under general anesthesia. Two or three incisions were opened during the operation. At first, a 10 mm camera port was placed on the 7th and 8th intercostal space of the mid-axillary line, and a 30–40 mm incision was made in the 4th and 5th intercostal space of the anterior axillary line. For difficult surgeries, a 15 mm auxiliary port was placed in the 8th or the 9th intercostal space of the scapular line. The arteries and veins of the target segments were carefully searched according to the preoperative plan prepared based on the 3D-CT images or 3D printed models during the operation to avoid accidental damages. In the 3D-CT group, the reconstructed image is placed on another screen, and the circuit nurse helps the operator adjust the angle to observe the anatomy of segmental vessels and bronchus. In the 3D printing model group, a personalized 3D printed model was placed in front of a thoracoscopic screen in the operating room so that the operator could observe the anatomy of segmental vessels and bronchi at any time, which give surgeons cross-reference to perform the surgery. The lung expansion-collapse method was utilized to find the segmental boundaries. The target bronchus was then clamped, and the lung lobes were expanded to ensure that the correct bronchus was targeted. After that, the target bronchus was removed to completely expand the lung tissues. The lung at the contralateral side was ventilated for about 15 min, the target lung segment on the affected side was expanded, and the other lung segments were collapsed. According to the boundaries between the inflated target lung tissues and the collapsed lung tissues, the target lung segments were resected. The surgical margin of all malignant nodules must be no less than 2 cm or at least equal to the diameter of the tumor mass. Otherwise, thoracotomy should be performed if necessary.

### Data Analysis

General clinical data and perioperative indicators were collected. The former included patients’ gender, age, tumor size, and pathological type as well as whether patients were complicated with other diseases before operation. The latter included operation time, intraoperative blood loss, intraoperative input, intraoperative fluid output, drainage volume within 24 h post-operation, drainage volume within 48 h post-operation, postoperative chest tube duration, postoperative hospital stay, and complications, such as air leakage (>6 days), pneumonia, atelectasis, and hemoptysis. In addition, the number of cases that had to be converted to thoracotomy due to any difficulties in surgery was also counted.

### Statistical Analysis

Data were analyzed using SPSS 26.0 (IBM Corporation, Armonk, NY, USA). Continuous variables were expressed as mean ± standard deviation (SD). Categorical variables are expressed as frequency and percentage. Differences in continuous variables between the two groups were analyzed using independent-sample t-test and Mann-Whitney U analysis. Differences in the incidence of postoperative complications between the two groups were analyzed using χ^2^ or Fisher's exact test. A *p* value <0.05 was considered statistically significant.

## Results

In case 1, the lung cancer was located in S1 of the left lung, which was close to relative pulmonary artery (A1 +2 ), vein (V1 + 2) and bronchi (B1 + 2). B3 was a separate branch, and B1 and B2 were from the same trunk (**Figure 2**). In case 2, the lung cancer was located in S1 of the right lung, which was close to relative pulmonary artery (A1), vein (V1) and bronchi (B1). B1 was a separate branch. B2 and B3 were from the same trunk. The topographic anatomy of artery was similar to the corresponding bronchi (**Figure 3**).

**Table 1** summarizes the general clinical data of the two groups of patients. There were more patients with hypertension before surgery in the 3D printing group than in the 3D-CT group. The gender, age, tumor size, pathological type, and the number of patients with diabetes or heart diseases before surgery were not significantly different between the two groups. **Table 2** shows the location of the resection of each lung segment and the number of cases. **Table 3** shows that (1) the intraoperative fluid input (1,158.5 ± 290.2 mL) for patients was significantly less than that in the 3D-CT group (1,433.2 ± 653.3 mL, *P* = 0.013); (2) the intraoperative fluid output for patients in the 3D printing group(731.0 ± 409.3 mL) was significantly less than that in the 3D-CT group (944.5 ± 500.3 mL, *P* = 0.012); (3) the operation time for patients in the 3D printing group, (162.7 ± 47.0 min)was also significantly shorter than that in the 3D-CT group (190.3 ± 56.9 min, *P* = 0.006). The intraoperative blood loss, 24 h pleural fluid volume, 48 h pleural fluid volume, postoperative chest tube duration, and postoperative hospital stay were not significantly different between the two groups (*P* > 0.05). One case in the 3D printing group and 2 cases in the 3D-CT group were converted from VATS segmentectomy to thoracotomy. The cause for the conversion of the two cases in the 3D-CT group was heavy hemorrhage due to intraoperative damages to the arteries, and that for the one case in the 3D printing group was the difficulty in effective removal of the lymph nodes to avoid excessive involvement of the force to damage the blood vessels. The incidence of complications was not significantly different between the two groups (*P* > 0.05). In general, if one lung ventilation is difficult to maintain and local exposure is difficult; the portal nail lymph node makes it difficult to dissect the blood vessels of the lung segment; bleeding that is difficult to control under endoscopy, we will choose to open a small auxiliary incision for treatment. In addition, we think that preserving more lung tissue and function as much as possible will be better helpful to the quality of life of patients in the future. Therefore, although we have opened an auxiliary incision, we also choose to continue segmentectomy and avoid lobectomy.

**Table 1:** Clinical characteristics of patients in 3D-CT and 3D printing groups.

Characteristics	3D-CT group (*n* = 57)	3D printing (*n* = 61)	*P*
Age (mean ± SD), yrs	53.1 ± 11.7	56.2 ± 10.6	0.134
Sex, *n* (%)			
Male	19 (33.3)	23 (37.7)	0.620
Female	38 (66.7)	38 (62.3)	
Tumor size, mm	12.4 ± 6.3	13.3 ± 8.4	0.695
Pathology, *n* (%)			
NSCLC	41 (71.9)	49 (80.3)	0.284
Benign	16 (28.1)	12 (19.7)	
Comorbidity, *n* (%)			
Hypertension	0 (0)	9 (14.8)	0.003
Diabetes mellitus	1 (1.8)	5 (8.2)	0.208
Heart diseases	2 (3.5)	2 (3.3)	1.000

**Table 2:** Segmentectomy position and number of cases in 3D-CT and 3D printing groups.

Segmentectomy position	3D-CT group (*n* = 57)	3D printing (*n* = 61)
LS1 + 2	7	2
LS1 + 2 + 3	5	3
LS3	2	1
LS4 + 5	5	5
LS6	5	11
LS8	2	0
LS10	1	0
LS9 + 10	1	1
RS1	5	5
RS2	4	13
RS3	7	2
RS1 + 3	2	0
RS1 + 2	2	0
RS2 + 3	1	0
RS6	4	13
RS6 + 10	1	0
RS7 + 8	1	2
RS8	1	2
RS8 + 9	1	1

**Table 3:** Intraoperative and postoperative data of patients in 3D-CT and 3D printing groups.

Variables	3D-CT (*n* = 57)	3D printing (*n* = 61)	*P*
Intraoperative blood loss (mean ± SD), mL	144.1 ± 143.1	140.5 ± 85.0	0.300
Intraoperative fluid input, mL	1,433.2 ± 653.3	1,158.5 ± 290.2	0.013
Intraoperative fluid output, mL	944.5 ± 500.3	731.0 ± 409.3	0.012
24 h pleural fluid volume, mL	212.0 ± 151.6	290.0 ± 256.3	0.243
48 h pleural fluid volume, mL	248.6 ± 349.3	250.2 ± 170.6	0.072
Operation time (mean ± SD), min	190.3 ± 56.9	162.7 ± 47.0	0.006
Postoperative chest tube duration (mean ± SD), day	4.7 ± 2.5	4.9 ± 1.9	0.257
Postoperative hospital stays, day	7.2 ± 2.6	6.7 ± 2.1	0.344
Conversion from VATS segmentectomy to thoracotomy or lobectomy, *n* (%)	2 (3.5)	1 (1.6)	0.609
Postoperative complications, *n* (%)			
Pulmonary air leakage (>5 days)	5 (8.7)	2 (3.2)	0.383
Hemoptysis	1 (1.8)	1 (1.6)	1.000
Pneumonia	10 (17.5)	8 (13.1)	0.504
Atelectasis	1 (1.8)	1 (1.6)	1.000

## Discussion

The standard surgical procedure for the treatment of NSCLC is lobectomy plus lymph node dissection (2) and segmentectomy is only used for patients with poor cardiopulmonary function or patients who were unable to withstand lobectomy. However, many studies have shown that for NSCLC smaller than 2 cm, there is no statistical difference between segmentectomy and lobectomy in overall survival rate and postoperative complications (3, 12, 13). Therefore, more and more hospitals choose segmentectomy to treat early NSCLC.

3D printing is a modern technology developed in recent years. The advantage of 3D printing is that it can more intuitively observe the tumor's location and relationship with the surrounding important blood vessels, avoiding intraoperative bleeding. 3D printing is now used in many clinical disciplines and is considered to have certain clinical values (6, 7). In recent years, personalized 3D printed models have also shown unique advantages in complex thoracic surgeries (14–17). Our results showed that the operation time of the 3D printing group (162.7 ± 47.0 min) was significantly shorter than that of the 3D-CT group (190.3 ± 56.9 min). The difference in operation time was mainly caused by complications that occurred during locating lung nodules and separating blood vessels. In the 3D printing group, intraoperative lung nodules locating is faster and more accurate, which is consistent with the results of Cheng et al. (18). In some patients, the pulmonary artery and pulmonary vein overlap in 3D-CT, which affects the judgment of the surgeon and prolongs blood vessel separation, although this only occurs in a small portion of patients (4). The anatomical variation of the pulmonary blood vessels not only prolongs the operation time but is also an important reason for the conversion to thoracotomy. Although 3D-CT can solve this problem better, there are still a small number of anatomical variations that cannot be identified on 3D-CT. Our data shows that 2 cases in the 3D-CT group were converted to thoracotomy due to heavy hemorrhage caused by damaged arteries, while one case in the 3D printing group was converted to thoracotomy because the lymph nodes were too hard to be effectively cleaned up and to avoid nearby blood vessels rupture caused by too much force to be involved in the lymph nodes. No patient in the 3D printing group was converted to thoracotomy because of vascular damage. Moreover, 3D printing technology can effectively identify variant blood vessels and prevent hemorrhage due to blood vessel damages during the operation. All these indicate that 3D printing can guide the operation more quickly, smoothly, and safely.

There are more hypertensive patients in the 3D printing group before the surgery, which means that patients in the 3D printing group had worse physical conditions than patients in the 3D-CT group. However, the 24 h pleural fluid volume, 48 h pleural fluid volume, postoperative retention time, and postoperative pleural fluid volume, hospital stay, and postoperative complications were not statistically significant between the two groups. This might be related to the fact that 3D printed models can identify arteries, veins, and bronchi faster than 3D-CT, reduce excessive tissue separation, avoid more surgical damages, and perform operations faster and more smoothly. The intraoperative fluid input and output were also significantly less in the 3D printing group than in the 3D-CT group (1,158.5 ± 290.2 mL vs. 1,433.2 ± 653.3 mL, and 731.0 ± 409.3 mL vs. 944.5 ± 500.3 mL respectively), which are conducive to the postoperative recovery of patients and preventing the emergence of postoperative complications. Most of the differences between the two types of surgical planning (fluid output and input, urine excretion) are related to surgery duration. Shorter operation time can reduce the incoming and outgoing volume during operation, which is of great significance to the patients’ perioperative rehabilitation, reduce complications and economic costs.

Moreover, studies have shown that patients who received 3D printing models before surgery could better understand the surgical process and the risks of surgery, which can help reduce the risk of doctor-patient conflict to a certain extent (19). In this study bleeding volume of patients in the 3D printing group and the 3D-CT group in this study was not significantly different. Because this study covered our some early surgical cases and some cases with more bleeding, the average bleeding volume is about 140 mL. At present, with the maturity of surgical technology, the amount of bleeding in our segmentectomy was generally less than 50 mL. Although the postoperative hospital stay and the incidence of complications were less in the 3D-CT group than in the 3D-CT group, the differences were not significant (*p* > 0.05), possibly due to small sample size. We will expand the sample size later to obtain more comprehensive and accurate results. The cost, time and easy waste of resources of making models are indeed the disadvantages of 3D printing. Our research group is proficient in 3D printing technology and can make qualified models in 2–3 days. In addition, we can reduce costs and save resources by printing only the lung segments involved in the operation and using cheap materials.

In summary, this study shows that in the preoperative planning of thoracoscopic segmentectomy, the 3D printing group had a shorter operation time and less intraoperative fluid input and output than the 3D-CT group. Compared with 3D-CT, 3D printing maybe guide the operation more safely and effectively.

## Data Availability

The original contributions presented in the study are included in the article/Supplementary Material, further inquiries can be directed to the corresponding author/s.
